# Early Venous Filling Following Thrombectomy: Association With Hemorrhagic Transformation and Functional Outcome

**DOI:** 10.3389/fneur.2021.649079

**Published:** 2021-03-10

**Authors:** Sophie Elands, Pierre Casimir, Thomas Bonnet, Benjamin Mine, Boris Lubicz, Martin Sjøgård, Noémie Ligot, Gilles Naeije

**Affiliations:** ^1^Department of Neurology, Erasmus Hospital, Université Libre de Bruxelles, Brussels, Belgium; ^2^Department of Interventional Neuroradiology, Erasmus Hospital, Université Libre de Bruxelles, Brussels, Belgium; ^3^Laboratoire de Cartographie Fonctionnelle du Cerveau, Neuroscience Institute (ULB-Neuroscience Institute), Université Libre de Bruxelles, Brussels, Belgium

**Keywords:** acute stroke, angiography digital subtraction, early venous filling, cerebral hemorrhage, thrombectomy, reperfusion after ischemia

## Abstract

**Background and Purpose:** Previous studies have noted the angiographic appearance of early venous filling (EVF) following recanalisation in acute ischemic stroke. However, the prognostic implications of EVF as a novel imaging biomarker remain unclear. We aimed to evaluate the correlation between EVF with (i) the risk of subsequent reperfusion hemorrhage (RPH) and (ii) the association of EVF on both the NIHSS score at 24 h and functional outcome as assessed with the Modified Rankin Scale (mRS) score at 90 days.

**Methods:** We conducted a retrospective cohort study of patients presenting with an acute ischemic stroke due to a proximal large-vessel occlusion of the anterior circulation treated by thrombectomy. Post-reperfusion digital subtraction angiography was reviewed to look for EVF as evidenced by the contrast opacification of any cerebral vein before the late arterial phase.

**Results:** EVF occurred in 22.4% of the 147 cases included. The presence of EVF significantly increased the risk of RPH (*p* = 0.0048), including the risk of symptomatic hemorrhage (*p* = 0.0052). The presence of EVF (*p* = 0.0016) and the absence of RPH (*p* = 0.0021) were independently associated with a better outcome as defined by the NIHSS difference at 24 h, most significantly in the EVF^+^RPH^−^ group. No significant relationship was however found between either EVF or RPH and a mRS score ≤ 2 at 90 days.

**Conclusion:** Early venous filling on angiographic imaging is a potential predictor of reperfusion hemorrhage. The absence of subsequent RPH in this sub-group is associated with better outcomes at 24 h post-thrombectomy than in those with RPH.

## Introduction

Stroke is the second most common cause of death and the main cause of acquired disability worldwide (1). Over sixty percent of morbidity and mortality related to stroke is due to large vessel occlusion (LVO) (2), which in itself accounts for about 30% of all ischemic strokes (3). The primary therapeutic aim is to rapidly recanalize the occluded vessel in order to restore blood flow and salvage cerebral tissue so as to improve patient outcome. In that context, endovascular thrombectomy (EVT) with or without intravenous thrombolysis substantially reduces disability in selected cases of LVO (4). The benefit of recanalizing treatments must be balanced with procedural risks and LVO stroke complications such as hemorrhagic transformation and reperfusion hemorrhage (RPH), with hemorrhagic transformation occurring in up to 43% of patients (5). These hemorrhagic complications tend to be classified based on their radiological appearance according to the European Cooperative Acute Stroke Study (ECASS II) into parenchymal hematomas (PH) and hemorrhagic infarctions (HI). The incidence of PH after EVT was recently reported to be 6% (6), with PH strongly correlating with early neurological deterioration and poor clinical outcome (7).

Although time is of the essence in achieving recanalization, there has been a recent paradigm shift whereby neuroimaging is gaining center stage in EVT decision-making. It provides an invaluable insight that is both patient-specific and dynamic into the physiological effects of the vessel occlusion, the penumbra at stake and RPH risks. Neuroimaging thus plays a key role in providing a tailored-made risk-benefit calculation for recanalization intervention and prediction of treatment response (8).

Current pre-treatment evaluation techniques include perfusion imaging derived from either computer tomographic (CT) or magnetic resonance imaging (MRI), which allow a quantitative assessment of cerebral blood flow (CBF), cerebral blood volume (CBV), and mean transit time (MTT). These measures help to evaluate the degree of salvageable “penumbra,” in other words the area of brain tissue surrounding the irreversibly damaged “infarcted core” that is at risk of infarction but may still be saved if reperfused. As such, the infarcted core is usually defined on CT as a CBF <30% of normal brain blood flow or on MRI as an apparent diffusion coefficient <620 μm^2^/s, whereas the area of critical hypoperfusion is identified as MTT of >6 s. The estimated penumbra, otherwise known as mismatch volume, is derived from the difference between these two values. However, in LVO, EVT decision currently relies on perfusion characteristics only when symptom onset exceeds 6 h. There, a favorable mismatch allows to extend the therapeutic window to as far as 24 h post-symptom onset (8). Similarly, perfusion imaging helps to assess the risk of bleeding following EVT, with an increased risk of hemorrhagic transformation in cases with a large ischemic core volume, severe blood flow restriction, blood-brain barrier disruption and poor collateral status (9).

However, within 6 h of symptoms onset in LVO, perfusion imaging is not warranted, preventing its use as prognostic tool for clinical outcome or complications in most of cases. In that context, digital subtraction angiography (DSA) could provide valuable information. As such, there is scarce evidence about the post-recanalization imaging biomarkers available on digital subtraction angiography (DSA). Prominent brain vascularity in the form of capillary blush, arteriovenous shunting and early venous filling (EVF) have been noted immediately after EVT (9). EVF, defined as the contrast opacification of any cerebral vein before the late arterial phase on post-reperfusion DSA, has previously been shown to be associated with an increased risk of subsequent infarction (10–12), a higher rate of reperfusion hemorrhage (RPH) and worse clinical outcomes (10, 13). However, these findings were limited by either outdated recanalisation techniques or small cohort size.

Here, we investigated the association between EVF and RPH, together with its impact on functional prognosis and physiopathological correlations by conducting a retrospective study on the largest cohort to date of patients undergoing thrombectomy for a proximal anterior circulation occlusion.

## Materials and Methods

The data that support the findings of this study are available from the corresponding author upon reasonable request.

### Patient Cohort

Ethics approval was obtained from our local institutional review board to conduct a retrospective cohort study with data collected from the medical records of patients presenting at Erasmus Hospital in Brussels with an acute ischemic stroke treated by thrombectomy between December 2014 and September 2019. Inclusion criteria were patients presenting with (1) a proximal occlusion of the anterior circulation including the internal carotid artery, M1 or M2 divisions of the Middle Cerebral Artery (MCA), (2) baseline Modified Rankin Scale (mRS) score of ≤ 2, (3) treated by mechanical thrombectomy within 24 h of symptom onset, (4) with a reperfusion score of Thrombolysis in Cerebral Infarction (TICI) equal or > II, and (5) imaging by CT within 24 h and/or MRI within 7 days of revascularization. The procedures followed were in accordance with institutional guidelines. Exclusion criteria included those with missing clinical data for statistical analysis.

### Imaging

**Figure 1:**
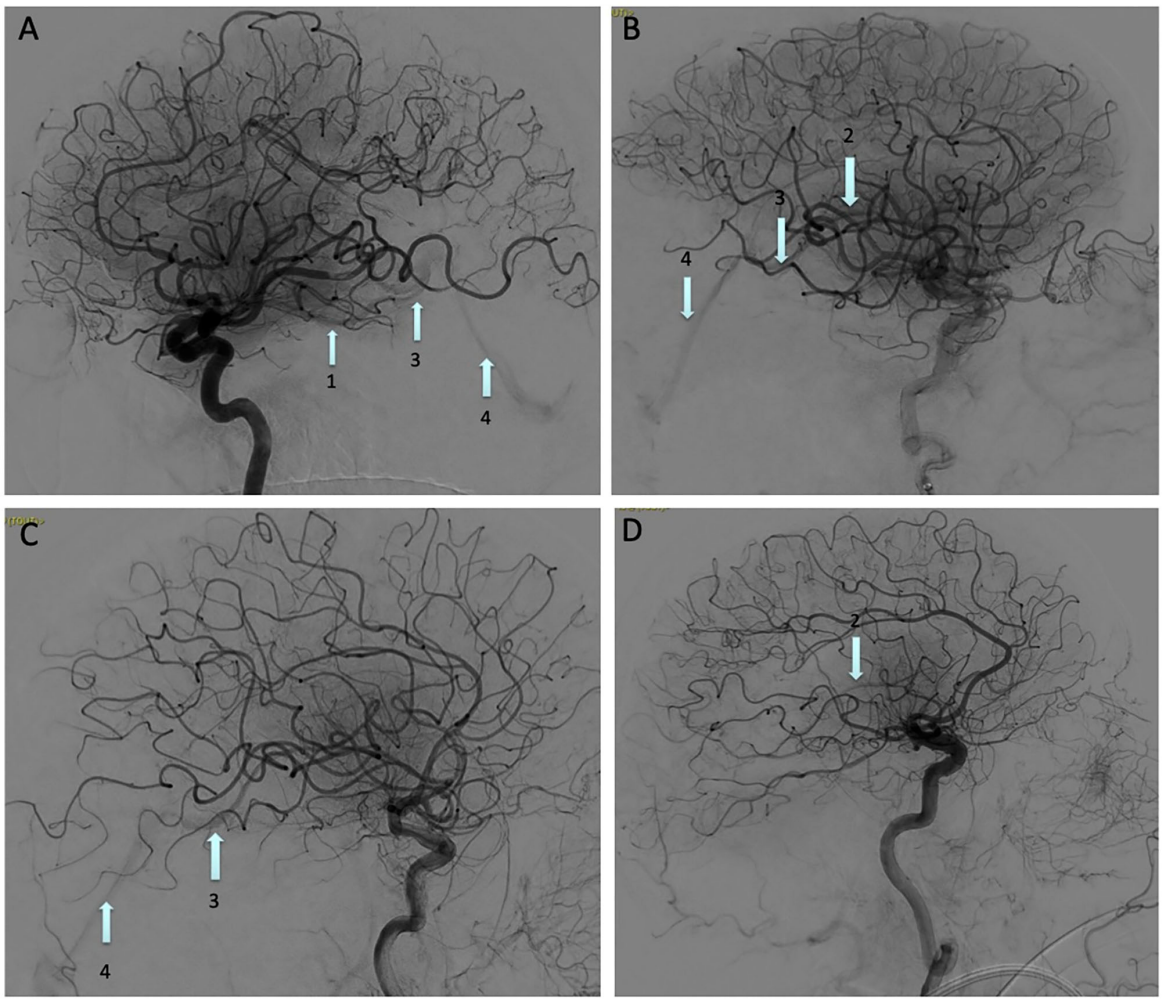
Digital subtraction angiography images showing an Early Venous Filling (EVF) following recanalization of: **(A)** M1 occlusion, **(B)** M2 temporal branch occlusion, **(C)** M1 occlusion, and **(D)** Terminal portion of the internal carotid artery occlusion. Basal vein of Rosenthal (arrow 1), internal cerebral vein (arrow 2), great vein of Galen (arrow 3), straight sinus (arrow 4).

Pre-interventional imaging included non-contrast CT and CT angiography. Additional CT perfusion imaging was done if the patient presented >6 h after symptom onset as per current guidelines. Occasionally MRI with FLAIR, time-of-flight MR angiography, as well as diffusion and perfusion imaging were used if there was a contra-indication to CT imaging. Early Venous Filling (EVF) is defined as the contrast opacification of any cerebral vein before the late arterial phase on post-reperfusion DSA and rated as either present or absent (Figure 1 depicts four illustrative cases). Reperfusion hemorrhage (RPH) was noted on CT or MRI, and classified based on their radiological appearance according to the European Cooperative Acute Stroke Study (ECASS II) into hemorrhagic infarction (HI) and parenchymal hematoma (PH), with HI1 defined as small petechiae along the ischemic margins, HI2 as confluent petechiae within the infarcted zone, PH1 as blood clots in <30% of the ischemic area with mild mass effect, and PH2 as blood clots in >30% of the ischemia zone with marked mass effect (5). A symptomatic hemorrhage was defined as RPH associated with an increase in NIHSS score of >2. All imaging was reviewed by two independent interventional neuroradiologists blinded to clinical outcome without concertation. EVF was considered present when identified by both neuroradiologists.

### Outcomes

The primary outcome measure was the occurrence of RPH, recorded as present or absent. The secondary outcome measure was the functional outcome, using (1) the National Institutes of Health Stroke Scale (NIHSS) score difference between hospital admission and 24 h post-thrombectomy, and (2) the Modified Rankin Scale (mRS) score at 90 days with a good clinical outcome defined as a score ≤ 2.

### Statistical Analysis

To determine whether EVF status was significantly related to the probability of (i) reperfusion hemorrhage and (ii) symptomatic hemorrhage, we used independent multiple logistic regression models with RPH or symptomatic hemorrhage as the dependent variables and EVF as regressor alongside the following potential confounding variables—age, sex, NIHSS score at admission, time to recanalization, site of occlusion and the presence of prior antiplatelet or anticoagulant therapy—to control for the effect of these on the EVF-RPH relationship.

In order to investigate the relationship between the functional outcome and (i) EVF and (ii) RPH, we included both in separate regression analyses with the two functional outcomes as the dependent variable in separate models. Specifically, we performed a multiple linear regression to investigate the relationship between the NIHSS difference (between admission and 24 h post-thrombectomy) and the regressors EVF and RPH, whilst also controlling for the other confounding variables listed above. In addition, we performed the same analysis using symptomatic RPH. Similarly, we performed a multiple logistic regression analysis to investigate the relationship between the binarized mRS outcome (with a good outcome being a mRS ≤ 2) as dependent variable and the same regressors listed above.

To investigate whether the presence of symptomatic hemorrhage or any of the subtypes of RPH were found in significantly different proportions in the EVF^+^ and EVF^−^ groups, we conducted five independent chi square tests for the difference in proportion of HI1, HI2, PH1, PH2, and symptomatic hemorrhage between the groups.

## Results

**Figure 2:**
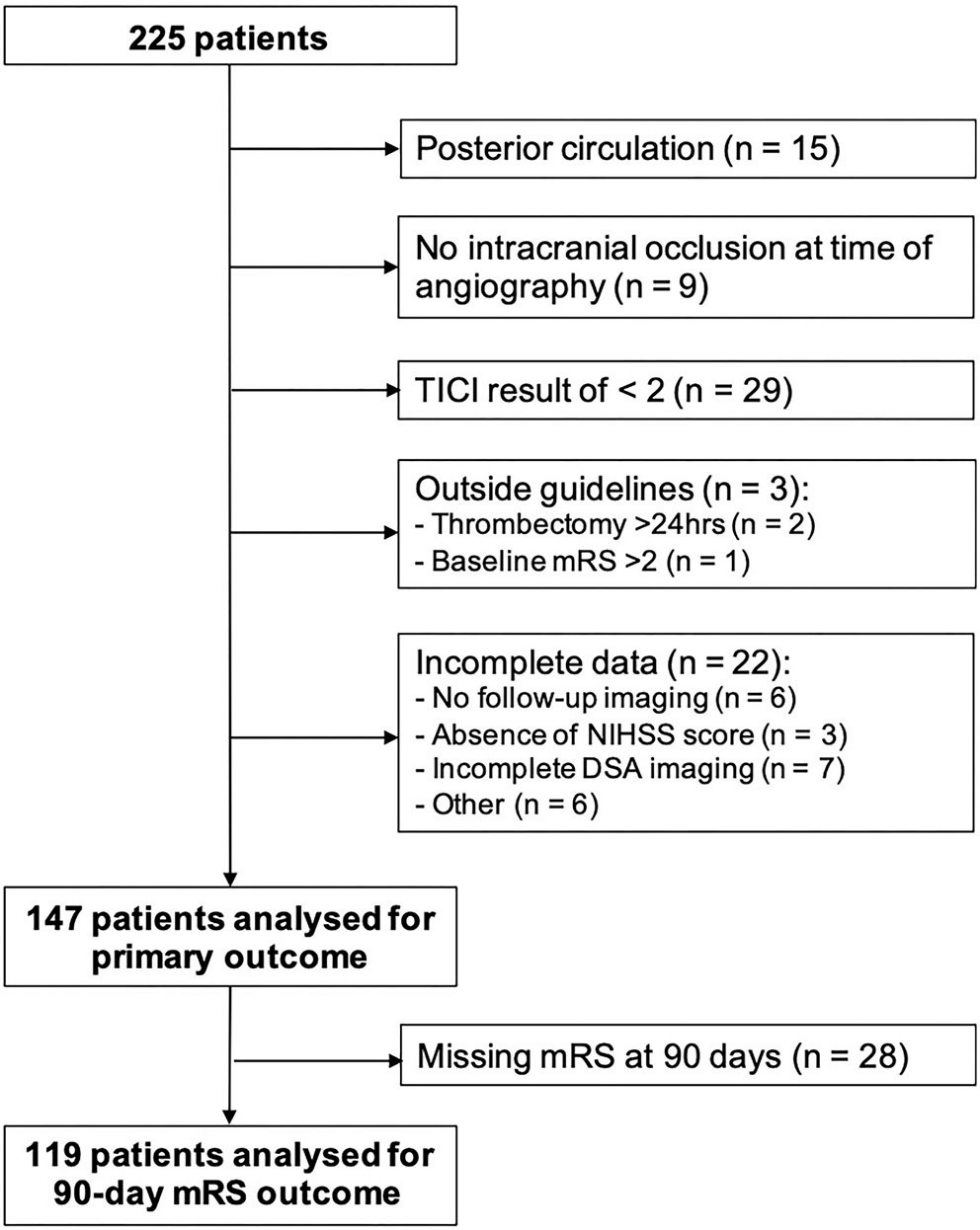
Flow diagram of patients in this study.

We identified 225 patients who underwent a thrombectomy. Seventy-eight met exclusion criteria (Figure 2), yielding a total of 147 patients. Seven patients had missing mRS scores at 90 days (4 EVF^−^RPH^−^ and 3 EVF^+^RPH^−^), so only 140 patients were included in the analysis of these outcomes.

Of the 147 cases, 39 and 33 cases were noted to have an EVF, respectively, by neuroradiologist 1 (BM) and neuroradiologist 2 (TB) of which 33 were congruent and considered to have definite EVF (22.4%) (Cohen Kappa coefficient for EVF 0.23, 66% of agreement). The baseline clinical characteristics of both groups (EVF^−^ and EVF^+^) are summarized in Table 1. There was no significant difference between these groups in terms of demographics, cardiovascular risk factors, prior antiplatelet/anticoagulation treatment, pre-therapeutic NIHSS, occlusion site, door-to-needle time or TICI result.

**Table 1:** Clinical characteristics.

**Characteristics**	**All (%)**	**EVF^**−**^ (%)**	**EVF^**+**^ (%)**
Number of patients	147	114 (77.6)	33 (22.4)
Age (average)	72.8	71.2	78
Female	101 (68.7)	79 (69.3)	22 (66.7)
Hypertension	105 (71.4)	79 (69.3)	26 (78.8)
Hypercholesterolemia	79 (53.7)	57 (50)	22 (66.7)
Diabetes	24 (16.3)	20 (17.5)	4 (12.1)
Coronary artery disease	43 (29.3)	33 (28.9)	10 (30.3)
Atrial fibrillation	66 (44.9)	48 (42.1)	18 (54.5)
Chronic kidney disease	24 (16.3)	17 (14.9)	7 (21.2)
Smoking (Prior/Active)	49 (33.3)	43 (37.7)	6 (18.2)
Prior stroke	18 (12.2)	14 (12.3)	4 (12.1)
Prior intracerebral hemorrhage	3 (2.0)	3 (2.6)	0 (0)
**Anticoagulant/Antiplatelet therapy**			
Aspirin	48 (32.7)	39 (34.2)	9 (27.3)
Clopidogrel	7 (4.8)	6 (5.2)	1 (3.0)
Warfarin	13 (8.8)	10 (8.8)	3 (9.1)
Heparin	1 (0.7)	1 (0.9)	0 (0)
Direct oral anticoagulant	16 (10.9)	14 (12.3)	2 (6.1)
**Baseline scores**			
Baseline NIHSS (average)	15.5	15.0	17.2
Baseline mRS (average)	0.5	0.5	0.5
**Thrombolysis**			
IV tPA	36 (24.5)	26 (22.8)	10 (30.3)
Time to IV tPA (min), average	110	110	111
**Occlusion site**			
Internal carotid artery	49 (33.3)	35 (30.7)	14 (42.4)
M1	81 (55.1)	66 (57.9)	15 (45.5)
M2	17 (11.6)	13 (11.4)	4 (12.1)
**TICI score**			
2a	15 (10.2)	8 (7.0)	7 (21.2)
2b	52 (35.4)	41 (36.0)	11 (33.3)
3	80 (54.4)	65 (57.0)	15 (45.5)
Time to recanalisation (min), average	293	291	299
**Post-treatment imaging modality**			
CT	145 (98.6)	113 (99.1)	32 (97.0)
MRI	93 (63.3)	70 (61.4)	23 (69.7)
Both	92 (62.3)	70 (61.4)	22 (66.7)
**Reperfusion hemorrhage**			
Any hemorrhage	42 (28.6)	28 (24.6)	14 (42.4)
HI1	13 (8.8)	8 (7.0)	5 (15.2)
HI2	7 (4.8)	6 (5.3)	1 (3.0)
PH1	15 (10.2)	10 (8.8)	5 (15.2)
PH2	7 (4.8)	4 (3.5)	3 (9.1)

*EVF, Early Venous Filling; NIHSS, National Institutes of Health Stroke Scale; mRS, modified Rankin Scale; IV tPA, Intra-Venous tissue Plasminogen Activator; TICI, Thrombolysis in Cerebral Infarction; CT, Computed Tomography; MRI, Magnetic Resonance Imaging; HI, Hemorrhagic Infarction; PH, Parenchymal Hematoma*.

Of those with RPH, 6 were symptomatic in the EVF^−^ group (3 PH1 and 3 PH2) compared to 1 PH2 in those EVF^+^. Neither symptomatic hemorrhage nor any of the four types of RPH (HI1, HI2, PH1, PH2) were found in significantly different proportions in the EVF^+^/EVF^−^ groups (χ^2^ <1.21, *p* > 0.271).

In the primary outcome analysis, the risk of RPH was significantly increased by the presence of EVF (odds ratio 6.68, *p* = 0.0048) when controlling for other confounding factors. Similarly, the presence of EVF significantly increased the risk of *symptomatic* RPH (odds ratio 5.8, *p* = 0.0052).

With regards to the secondary outcome, a multiple regression analysis (R^2^ = 0.153, *F* = 4.82, *p* = 8.0 × 10^−5^) showed that the NIHSS difference had a significant inverse relationship to EVF (β = −4.75, *t* = −2.93, *p* = 0.0016) and a significant positive relationship to the existence of RPH (β = 4.19, *t* = 2.87, *p* = 0.0021) when controlling for the known confounding factors. In other words, the presence of EVF was associated with an improvement (i.e., decrease) in NIHSS score independently of RPH, whereas the occurrence of RPH was associated with a worsening (i.e., increase) in NIHSS. The same analysis using the existence of a *symptomatic* RPH instead showed that this had an even more significant positive relationship with NIHSS difference (β = 13.57, *t* = 5.75, 6.56 × 10^−8^).

These regression analyses show that the presence of EVF (EVF^+^) and the absence of RPH (RPH^−^) are independently associated with a better outcome as defined by the NIHSS difference. In addition, there was no significant effect of the interaction between EVF and RPH on the outcome. For these reasons, we would expect the EVF^+^RPH^−^ group to be associated with the best outcome compared to the other three combinations, which is indeed what we observe (see Table 2).

**Table 2:** Functional outcomes by EVF and RPH subgroups.

**NIHSSdiff**	**EVF^**+**^**	**EVF^**−**^**	**mRS90**	**EVF^**+**^**	**EVF^**−**^**
RPH^+^	5.2	3.3	RPH^+^	3 (23.1%)	6 (20.1%)
RPH^**−**^	10.2	6.1	RPH^**−**^	10 (66.7%)	35 (51.5%)

*EVF, Early Venous Filling; RPH, Reperfusion Hemorrhage; NIHSSdiff, National Institutes of Health Stroke Scale difference at 24 h; mRS90, Modified Rankin Scale score at 90 days*.

In contrast, when looking at the mRS at 90 days, a multiple logistic regression analysis showed no significant relationship between mRS (binarized as either good mRS ≤ 2 or bad >2) and either EVF, RPH or symptomatic RPH, when controlled for the same confounding variables described previously (coefficient *p* > 0.061).

## Discussion

In our study, EVF occurred in almost 25% of cases and was associated with both increased rate of RPH and improved functional outcome at 24 h in those combining EVF and no RPH. This double-edged sword effect may reflect a state of hyperperfusion, beneficial in preserving the penumbra, but also nefarious by increasing blood flow to a brain area made more fragile by ischemia.

In comparison to other large cohorts of LVO ischemic strokes (14), ours had a relatively higher proportion of women 68.7% (vs. 47%), hypertension 71.4% (vs. 57.5%), hyperlipidemia 53.7% (35.2%), and atrial fibrillation 44.9% (32.9%). Thus, care must be drawn before extrapolating the findings to a larger population. However, EVF occurrence in 22.4% of successful EVT cases aligns with the incidence found in previous studies using similar definitions of EVF (10, 13).

EVF was associated with a higher risk of both RPH and symptomatic hemorrhage, which is in line with the seminal study by Ohta et al. (10) where the rate of RPH and symptomatic hemorrhage (defined as massive hematoma with neurological worsening) was 61.3 and 32.3%, respectively, in their EVF^+^ group. This was in the context of a prospective study on superselective local angiography via microcatheter before and during intra-arterial reperfusion therapy for acute MCA occlusion. In comparison to our study, the observed increased bleeding risk if EVF^+^ was confounded by the use of microcatheter injections and intra-arterial thrombolysis, both independently associated with RPH. Similarly, Cartmell et al. (13) identified EVF to be a strong predictor of symptomatic parenchymal hematoma associated with a NIHSS decline of at least 3 points following revascularization therapy, with a sensitivity and specificity of both 0.83, albeit in a relatively small cohort size of 59 patients, similar in demographics to our population.

Independently of RPH, prior studies have suggested EVF to not be associated with a significant NIHSS shift (13), and to carry a less favorable long-term outcome as defined by an mRS>2 at 90 days (10, 13). However, our study suggests that EVF is associated with an initial functional improvement in terms of NIHSS difference at 24 h, especially so for those with EVF but no subsequent RPH. However, this functional improvement does not seem to be reflected in the long-term as we found no significant relationship between EVF and a mRS ≤ 2 at 90 days, even if there was a trend for higher rate of mRS score <2 (52.6 vs. 40.7%) at 90 days in subjects with EVF. The lack of significant long-term improvement is probably explained by the limited sample of our cohort and by the fact that after the first week, most complications relate to systemic rather than cerebrovascular complications clouding initial improvement results (15).

We hypothesize that EVF reflects a state of hyperemia, otherwise known as “luxury perfusion” (16), that could account for the better outcome in some and RPH in others. Indeed, diagnostic angiographic reports of EVF date back to the 1950s where EVF was thought to relate to an increasing circulatory rate or vasodilatation through the infarcted area, thereby enabling contrast material to reach the venous system more rapidly, especially once the occlusion has been relieved (17). This zone of focal hyperemia seems to correspond to a higher cerebral blood flow (CBF) and a reduced mean transit time (18, 19). Although hyperemia may equally be seen in the perifocal border zone around infarcts as an *angiographic blush*, EVF seems to reflect the presence of an ischemic core. Indeed, several studies showed that EVF was a predictor for irreversible regional tissue damage and subsequent infarction despite successful recanalization (10), with an estimated sensitivity of 88–90.3% and specificity of 63–81% (11, 12). In contrast, the angiographic blush is probably in part due to the leaking out of vasodilating substances from the infarcted area.

Post-ischemic hyperemia following recanalisation has been observed in up to 30–40% of patients on different imaging modalities including CT perfusion (20), diffusion-perfusion MRI (21), as well as arterial spin labeled perfusion MRI (22–24). The hyperperfusion tends to be confined to the ischemic brain territory and is associated with an increased risk of hemorrhagic transformation (22–24), as well as an improved NIHSS score at 24 h and mRS ≤ 2 at 90 days (24). Similarly, PET imaging has demonstrated early focal hyperperfusion <48 h after onset may be a harmless and perhaps even a beneficial phenomenon (25, 26). These results parallel our findings and support EVF as an angiographic marker of hyperperfusion.

The exact pathophysiological basis of cerebral hyperemia is incompletely understood and probably multifactorial. Lassen (16) in 1966 suggested that an acute vessel occlusion may lead to focal hypoxia, with an increase in pCO_2_ and a lowering of the pH, resulting in the release of vasoactive substances that stimulate capillary dilatation. Others have postulated the opening of arteriovenous shunts that bypass blocked capillary beds (27). Both are likely to lead to an increased regional cerebral blood flow. This is accompanied by damage to the cerebrovascular endothelium and disruption of the blood-brain barrier (28), all contributing to a loss of cerebral autoregulation.

Better understanding could come from recent advances that enable perfusion measurements to be derived quantitatively from DSA data acquired during EVT, offering the possibility of a more dynamic visualization of the cerebral hemodynamics during an acute ischemic stroke. Using this technique, Kosior et al. (19) demonstrated focal areas of hyperperfusion in 10 out of 50 patients shortly after recanalisation with focal areas of hyperemia corresponding to perfusion maps with a short T_Max_ and MTT. A U-shaped relationship between MTT and RPH was found, with the risk of hemorrhage greatest at both the lowest and highest extremes of MTT, reflecting the phenomena of hyperperfusion and no-reflow at either end of the spectrum. Their findings were however limited due to the lack of data on patient outcome. Nevertheless, it would be interesting to correlate their perfusion measurements with EVF on DSA to determine whether it corresponds to these visualized areas of focal hyperemia, thereby supporting our hypothesis.

EVF may thus reflect a state of hyperperfusion localized to the ischemic territory. This may be beneficial in preserving the penumbra, thereby leading to a better short-term functional outcome. It may however also bear an increased risk of reperfusion hemorrhage by increasing blood flow to an area made more fragile by ischemic damage. These findings may have implications on subsequent blood-pressure control and the initiation of antiplatelet or anticoagulant therapy post EVT.

## Conclusion

In patients undergoing thrombectomy for a proximal anterior circulation occlusion, EVF is associated with both RPH and an improved NIHSS score at 24 h, reflecting a state of hyperperfusion localized to the ischemic territory. These findings bring new insights on post-ischemic cerebral blood flow regulation and have a potential impact on blood pressure management and decision-making around the timing of subsequent antiplatelet/anticoagulation treatment. EVF is thus a novel angiographic biomarker offering a personalized and dynamic insight into the individual patient's EVT treatment response.

## Data Availability Statement

The raw data supporting the conclusions of this article will be made available by the authors, without undue reservation.

## Ethics Statement

The studies involving human participants were reviewed and approved by Ethics Committee of Erasmus Hospital, Brussels, Belgium. Written informed consent for participation was not required for this study in accordance with the national legislation and the institutional requirements.

## Author Contributions

SE and GN designed the study. SE, PC, TB, and BM collected the data. SE, MS, and GN analyzed the data. SE wrote the manuscript. BL, NL, and GN revised the article critically. All authors approved the version to be published.

## Conflict of Interest

The authors declare that the research was conducted in the absence of any commercial or financial relationships that could be construed as a potential conflict of interest.
